# Targeting hunter distribution based on host resource selection and kill sites to manage disease risk

**DOI:** 10.1002/ece3.788

**Published:** 2013-10-01

**Authors:** Cherie J Dugal, Floris M van Beest, Eric Vander Wal, Ryan K Brook

**Affiliations:** 1 Department of Animal and Poultry Science, College of Agriculture and Bioresources, University of Saskatchewan51 Campus Drive, Saskatoon, SK, S7N 5E2, Canada; 2 Department of Bioscience, Aarhus UniversityFrederiksborgvej 399, Roskilde, 4000, Denmark; 3 Département de Biologie, Université de Sherbrooke2500 boul. de l'université, Sherbrooke, QC, J1K 2R1, Canada; 4 Department of Animal and Poultry Science & Indigenous Land Management Institute, College of Agriculture and Bioresources, University of Saskatchewan51 Campus Drive, Saskatoon, SK, S7N 5E2, Canada

**Keywords:** Bovine tuberculosis, chronic wasting disease, elk, hunter-kill, mortality, protected area, resource selection function, selection ratio

## Abstract

Endemic and emerging diseases are rarely uniform in their spatial distribution or prevalence among cohorts of wildlife. Spatial models that quantify risk-driven differences in resource selection and hunter mortality of animals at fine spatial scales can assist disease management by identifying high-risk areas and individuals. We used resource selection functions (RSFs) and selection ratios (SRs) to quantify sex- and age-specific resource selection patterns of collared (*n* = 67) and hunter-killed (*n* = 796) nonmigratory elk (*Cervus canadensis manitobensis*) during the hunting season between 2002 and 2012, in southwestern Manitoba, Canada. Distance to protected area was the most important covariate influencing resource selection and hunter-kill sites of elk (AICw = 1.00). Collared adult males (which are most likely to be infected with bovine tuberculosis (*Mycobacterium bovis*) and chronic wasting disease) rarely selected for sites outside of parks during the hunting season in contrast to adult females and juvenile males. The RSFs showed selection by adult females and juvenile males to be negatively associated with landscape-level forest cover, high road density, and water cover, whereas hunter-kill sites of these cohorts were positively associated with landscape-level forest cover and increasing distance to streams and negatively associated with high road density. Local-level forest was positively associated with collared animal locations and hunter-kill sites; however, selection was stronger for collared juvenile males and hunter-killed adult females. In instances where disease infects a metapopulation and eradication is infeasible, a principle goal of management is to limit the spread of disease among infected animals. We map high-risk areas that are regularly used by potentially infectious hosts but currently underrepresented in the distribution of kill sites. We present a novel application of widely available data to target hunter distribution based on host resource selection and kill sites as a promising tool for applying selective hunting to the management of transmissible diseases in a game species.

## Introduction

Human hunting influences animal movements, resource selection, and population dynamics of wildlife (Lindsey et al. 2007; Juillet et al. 2012). Most management-oriented research has traditionally focused on the evolutionary consequences of selective hunting on the population dynamics of wildlife (Coltman et al. 2003; Servanty et al. 2011; Rivrud et al. 2013). However, few studies have considered the direct and indirect implications of hunting for managing disease risk in wild populations (Wasserberg et al. 2009; Wild et al. 2011). Traditional strategies for disease control in wildlife include test and slaughter, vaccination, or culling the wildlife host population; however, these methods are difficult to fully implement, and the effectiveness in reducing pathogen transmission is questionable at best (Peterson et al. 1991; Harrison et al. 2010; Beeton and McCallum 2011). As such, wildlife managers are faced with the challenge of limiting the spread of disease among infectious wild animals (e.g., Cross et al. 2007).

Although seasonal hunting has been used to manage wildlife diseases in the past (e.g., Schmitt et al. 1997), it has typically been employed at coarse spatial scales through a licensing or quota system to reduce host population size (Conner et al. 2008). Because the spatial distribution or prevalence of diseases are rarely distributed evenly within wild populations (Schmitt et al. 2002; Miller and Conner 2005; Härkönen et al. 2007; Shury and Bergeson 2011), the distribution of hunters could be more effectively focused at fine spatial scales and aimed at specific sex and age classes (i.e., cohorts) with the greatest potential for disease transmission (Schmitt et al. 2002; Grear et al. 2006).

Spatial models that quantify risk-driven differences in host resource selection and hunter-kill sites can assist disease management by identifying specific high-risk areas that are regularly used by potentially infectious hosts during the hunting season but currently underrepresented in the distribution of kill sites. These high-risk areas can be used to inform management to adjust hunting where needed and consequently target individuals with the greatest potential for disease transmission (i.e., demographic classes within a population with the highest disease prevalence).

Diseases such as chronic wasting disease (CWD) and bovine tuberculosis (*Mycobacterium bovis*, TB) infect communities of animals including both wild and domestic. Any infected individual can act as vectors of the disease, with the potential of spreading the disease from an endemic region into a new area. In this study, our goal was to assess the risk of disease spread among wild populations, specifically nonmigratory elk *Cervus canadensis manitobensis* Millais populations based on sex- and age-specific tracking data and hunter-kill sites collected in the Greater Riding Mountain Ecosystem in southwestern Manitoba, Canada. We recognize the transmission of disease among wildlife and livestock also creates important risks for conservation and agriculture in this area, and management strategies aimed to control disease at the wildlife–agriculture interface have been examined in detail (Brook and McLachlan 2009; Brook et al. 2012).

In this system, hunting is the major source of mortality of elk and transmissible diseases with severe socioeconomic and ecological implications continue to threaten resident ungulates (Brook 2009; Brook et al. 2012), especially bovine TB and CWD. The aims of this study were to (1) quantify age- and sex-specific differences in elk resource selection patterns during the hunting season, (2) evaluate and predict hunter mortality in relation to multiple landscape features and (3) develop maps that identify high-risk areas for disease transmission, which can be used to optimize hunter distribution to improve disease monitoring and control programs.

## Material and Methods

### Study area

#### Description of the Riding Mountain region

**Figure 1:**
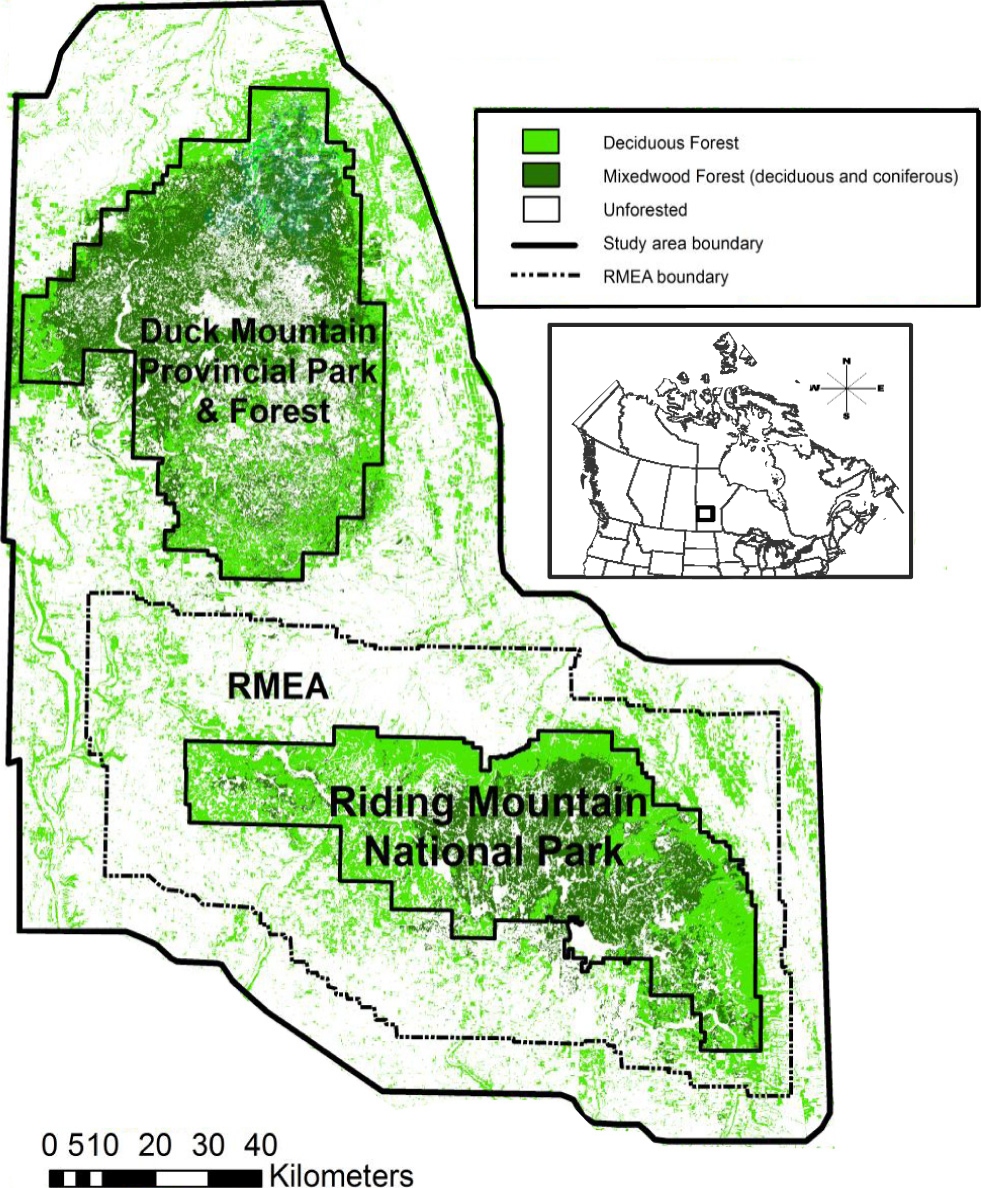
Map of the study area located on the agriculture-dominated lands surrounding Riding Mountain National Park and Duck Mountain Provincial Park and Forest in southwestern Manitoba, Canada. The Riding Mountain Eradication Area (RMEA) boundary is represented by the hashed line. Forest covariates were assessed using a 30-m spatial resolution map that was developed using Landsat 5 satellite imagery collected in 2003 and validated in 2011.

The Greater Riding Mountain Ecosystem (Fig. 1) is situated in a transition zone between the Prairies and the northern Boreal Plains ecozones in southwestern Manitoba, Canada (Wiken 1986). It includes the Riding Mountain Biosphere Reserve, an area designated as a zone of cooperation by the United Nations Educational, Scientific and Cultural Organization in 1986. The area consists of two protected areas: Riding Mountain National Park (RMNP; 2974 km^2^; 50°51′50″N, 100°02′10″W) and Duck Mountain Provincial Park and Forest (DMPP&F; 3756 km^2^; 51°39′58″N, 100°54′52″W). The surrounding agricultural landscape consisted of privately owned farmland (pasture and grain land; 70%), provincial (18%) and federal crown land (11%), and First Nation Reserves (1%; Brook 2008). The study area boundary is delimited by a 20-km buffer around the two large protected areas, but only includes the surrounding agricultural landscape and not the area within the parks. A regional population of ca. 2700 elk exist (Parks Canada and Manitoba Conservation, unpublished data) and remain largely within or near the forest-dominated protected areas, although individuals are known to use the adjacent farmland (Brook and McLachlan 2009; Brook et al. 2012).

#### Endemic and emerging disease in the Riding Mountain region

The Greater Riding Mountain Ecosystem includes the Riding Mountain Eradication Area (Fig. 1), which was created around RMNP in 2003 by the Canadian Food Inspection Agency in response to the rapid increase in bovine TB-infected cattle herds between 1991 and 2002 (Brook and McLachlan 2009; Brook et al. 2012; Vander Wal et al. 2012). Elk and white-tailed deer *Odocoileus virginianus* Zimmermann are both free-ranging hosts for bovine TB; however, the overall prevalence in elk has been found to be six times higher compared with white-tailed deer, suggesting elk may be a significant reservoir host for the disease in the area (Shury and Bergeson 2011).

Surveillance for bovine TB in the Riding Mountain Ecosystem was carried out within the period of 1997–2010, and prevalence has been examined in detail for all sex and age classes of wild elk (see Table 5 in Shury and Bergeson 2011). Shury and Bergeson (2011) report the overall elk population had a mean prevalence of 0.89% over the 12-year period, and a total of 41 positive elk were detected. There are significant differences in prevalence among RMNP elk: female prevalence was 0.70% and male prevalence was 1.17%. Differences were even more pronounced among age classes, with animals less than 1 year old having a prevalence of 0.22%, adults 6–8 years having 2.31%, and adults greater than 8 years having the highest prevalence of 2.57%.

Chronic wasting disease has not yet been detected in ungulates in Manitoba. However, CWD has emerged across the Canadian Prairies over the last decade as an important disease of concern in mule deer *O*. *hemionus* Rafinesque, white-tailed deer, and elk (Saunders et al. 2012). The study area is directly adjacent to the province of Saskatchewan, which has endemic CWD in wildlife (280 mule deer, 66 white-tailed deer, and four elk) and 66 captive cervid facilities (Canadian Cooperative Wildlife Health Centre and Saskatchewan Ministry of Agriculture, unpublished data). Establishment of endemic CWD in Alberta in 2005 resulted from infected wildlife from Saskatchewan moving across the border (Silbernagel et al. 2011). Recent evidence suggests that there are no barriers to prevent the movement of CWD-infected white-tailed deer into the Riding Mountain Region (Vander Wal et al. 2013). As such, there is a real risk of CWD spread into the region, which would cause considerable conservation and socioeconomic concerns.

#### Human hunting in the Riding Mountain region

Licensed and aboriginal subsistence hunting has been permitted since the early 1900s and has been known to affect the local ungulate populations (Green 1933). Licensed hunting is typically not permitted within the boundaries of the federal RMNP since its establishment in 1931; however, it is permitted within DMPP&F and on the agriculture matrix (defined here as the human-dominated area that surrounds both of these protected areas) during autumn and winter.

### Data sources

#### Collared elk data

Between 2002 and 2011, a total of 413 free-ranging elk were captured at random in and around RMNP and DMPP&F during the winter months (December to March) using a net gun fired from a helicopter and fitted with a global positioning system (GPS) satellite collar (24 F, 12 M), or a very high frequency (VHF) radio collar or ear transmitter (191 F, 186 M). An effort was made to capture as many individuals as possible on the surrounding agricultural lands outside of the parks. Locations of each GPS-collared animal were obtained daily (mean = 12 locations per day) for up to 2 years (Brook and McLachlan 2009). All GPS locations were screened for large positional outliers, and positions collected within 24 h of capture were excluded, which is typically carried out when assessing fine-scale animal movements (Bjørneraas et al. 2010). Locations of each VHF-collared animal were collected in and around the parks using fixed-wing aircraft and ground triangulation (mean = 3 locations every 2 weeks ± 1.4 locations) for up to 3.5 years (Brook 2008; Vander Wal et al. 2011).

We were interested in comparing collared elk locations with hunter-killed elk locations during the hunting season; therefore, only collared elk locations acquired during legal hunting hours from September to February were used (average 8:00–18:00 h). The effects of hunting can certainly influence elk movements beyond the hunting season months (Vieira et al. 2003) and cause shifts in their circadian patterns (Strohmeyer and Peek 1996); however, our approach was based on distribution in space rather than changing the distribution of the hunting season in time. As such, our application of hunting as a potential disease management tool can only be implemented during the hunting season and legal hunting hours. We also only selected animals with a home range that extended out of the parks to compare collared and hunter-killed locations on the agricultural matrix where hunting is allowed. As a result, these criteria reduced the final sample size to 16 GPS-collared animals from RMNP (*n =* 9 adult F; 5 adult M; 2 juvenile M) and 51 VHF-collared animals from RMNP (*n =* 33 adult F; 6 adult M; 12 juvenile M). For adult females, 57% were captured outside of RMNP, 9% for adult males and 14% for juvenile males. Elk were classified as adult female (≥2.5 year old), adult male (≥4 year old), and juvenile male (<4 year old; Flook 1970; Noyes et al. 1996). Juvenile female elk were not included in the analyses as they largely follow adult females and have the same selection patterns (Weckerly 1999).

#### Hunter-killed elk data

Between 2003 and 2012, a total of 796 hunter-killed animals were collected by the provincial wildlife management agency, Manitoba Conservation. Sex and age of the animal (estimated by tooth wear and antler growth; Keiss 1969), date and location of the kill were recorded. Each location was subsequently associated with the centroid of the quarter section whence it occurred. Quarter sections are 0.65 km^2^ units of land as defined by the Dominion Land Survey System (Richtik 1975). Land ownership, land management, and hunter access decisions are largely made at the scale of the individual quarter section within the study area; thus, the quarter section is the appropriate level for this analysis and consequent management actions. In this study, we assumed hunters to have been hunting near kill sites, and therefore, we also assume that higher density of kill sites corresponds to a combination of increased pressure and success. Hunter-killed elk were classified as adult female (≥2.5 year old), adult male (≥4 year old), and juvenile male (<4 year old; Flook 1970; Noyes et al. 1996). Kill sites were collected from September to February (*n =* 455 adult F; 135 adult M; 311 juvenile M; Manitoba Conservation and Parks Canada, 2003–2012, unpublished data).

#### Environmental covariates

A set of a priori environmental covariates was derived from the literature predicted to influence elk resource selection and hunter-kill sites during the hunting season (see Table S1 in Supporting Information). Habitat types included local-level forest cover within the quarter section and landscape-level forest cover within a 5-km buffer around the quarter section, grassland cover, annual cropland (oilseed and cereal), and perennial forage (hay and alfalfa). Water cover (lakes and rivers), distance to streams, and distance to protected area/park (DMPP&F and RMNP) were also included. Environmental covariates were measured at the level of the quarter section (*n* = 20,970) using an existing 30-m spatial resolution vegetation map that was developed using Landsat-5 satellite imagery collected in 2003 (Geobase; http://www.geobase.ca/) with ArcGIS 10 (ESRI Inc., Redlands, CA) and Geospatial Modelling Environment (Beyer 2012). Overall accuracy of the vegetation map was 84%, with the majority of map misclassification due to short-term changes in cropland.

Many covariates were measured on different scales; therefore, we standardized all covariates to a range of 0–1 for a more direct comparison. We also screened all covariates for correlations and collinearity using Spearman's rank correlation and variance inflation factors. When covariates were correlated or collinear (*r*_s_ ≤ 0.5 or VIF > 5), we removed the less significant. These included distance to town and distance to highway for collared juvenile males (*r*^2^ = 0.6), distance to highway was removed; and for hunter-killed juvenile males, cropland was removed (VIF > 5).

### Data analyses

In order to quantify age- and sex-specific differences in elk resource selection and predict hunter mortality in relation to multiple landscape features, we developed resource selection functions (RSFs) and resource selection ratios (SRs; Manly et al. 2002) for collared and hunter-killed elk separately. The presence or absence of locations was considered the dependant variable. We also calculated individual sets of SRs for adult female, adult male, and juvenile male elk for the period of the elk-hunting season. We excluded adult males in the RSF development due to a limited sample size of locations that were outside of protected areas. Resource use or a kill was based on individual quarter sections as sample units that contained either collared or harvested locations. We compared the variation of environmental covariates associated with the quarter section between used sections with an equal set of randomly generated available quarter sections (1:1 ratio) that were distributed throughout the entire study area using logistic regression (Boyce et al. 2002; Manly et al. 2002). As such, the analysis corresponded to that of second-order selection (Johnson 1980).

We used two separate but important modeling techniques used to predict the probability of elk use or a kill. We first developed a set of candidate a priori models to predict the probability of elk use or kill, using an information-theoretic approach (Burnham and Anderson 2002). Akaike's information criterion adjusted for sample size (ΔAIC_c_), and model weights (*w*_*i*_) were used to assess the fit of all models. We compared and ranked all a priori models according to their ability to explain both the probability of elk use and kill. We then used a multimodel inference approach as a separate modeling technique (independent of the a priori approach), based on all possible combinations of independent covariates, including 2-way interactions (Burnham and Anderson 2002; Whittingham et al. 2005). Results from the multimodel approach were used to develop sex-specific RSFs. We ranked all models based on a combination of covariates with the lowest ΔAIC_c_ for model inference using the Multi-Model Inference package in R (R Development Core Team 2012). All models with ΔAICc < 2.0 (Burnham and Anderson 2002) were retained, and resultant β coefficients and standard errors were used to derive the relative probability of elk use (elk collared data) or kill (hunter-killed elk locations) to produce RSFs maps. We rescaled all predicted RSF scores to a range of 0–1 for comparability and extrapolated across the entire study area. We evaluated the performance of the RSFs using the *k*-fold cross-validation procedure (Boyce et al. 2002) on the best model for both sexes by partitioning the data into five equal subsets. We then calculated cross-validated Spearman rank correlations (*r*_s_) between training and test data grouped within 10 bins.

We calculated the SRs for covariates from the best model of both sexes using the ratio of the proportion used to the proportion available:


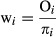


where O_*i*_ refers to the proportion of the *i*th covariate used at the collared or killed sites, and π_*i*_ represents the proportion available of that same covariate as determined by randomly generated locations throughout the study area, which is delimited by a 20-km buffer around the two large protected areas. The selection threshold is 1. If use of any given habitat is greater than its availability (i.e., selection is occurring), then SR > 1. If SR < 1, the habitat category is used less than available (i.e., avoided). If SR = 1, the habitat category is used as a function of its availability and is neither selected nor avoided.

To develop maps that identify high-risk areas for disease spread, we calculated the difference in RSF scores between collared and killed elk for both sexes. For example, one quarter section may have a predicted RSF score of 0.9 for collared elk (RSF scores range from 0 to 1) and a 0.2 for hunter-killed elk. This would indicate that the quarter section has a relatively high probability of elk use during the hunting season, but a low probability of a kill. The resultant RSF score would be 0.7, indicating that there remains a higher disease risk, as elk are not hunted as effectively in that quarter section. For an overall schematic outline of the analyses used to develop the disease risk management maps, see Figure S1 in Supporting Information.

## Results

### Resource selection functions

#### Collared elk

**Figure 2:**
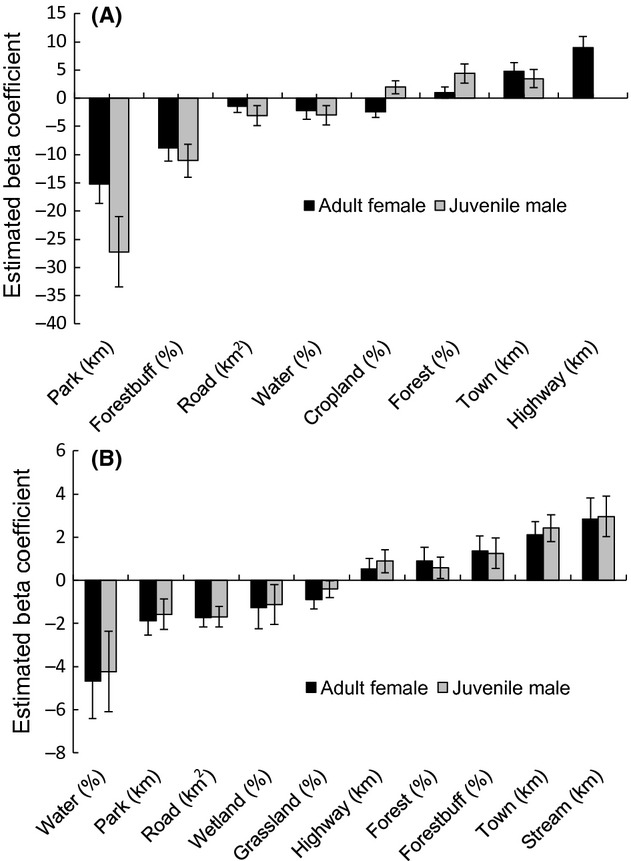
Model averaged coefficients (±SE) for covariates from logistic regression resource selection function models for (A) collared elk and (B) hunter-killed elk during the hunting season (September–February; 2002–2012) in southwestern Manitoba, Canada. Estimates were derived from an average of all possible models with a change in Akaike value < 2 (ΔAIC, 2.0).

**Figure 3:**
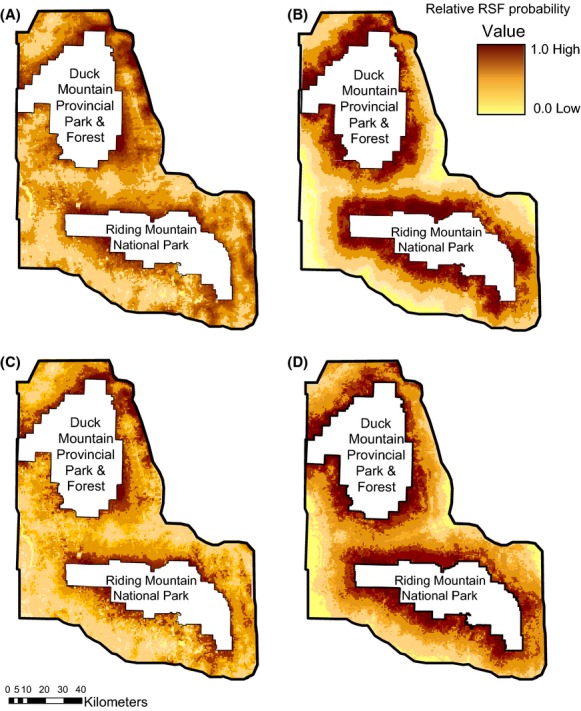
Interpolated map surfaces representing a resource selection function model for (A) collared adult females, (B) collared juvenile males, (C) hunter-killed adult females, and (D) hunter-killed juvenile males in southwestern Manitoba (September–February; 2002–2012). Darker shaded areas represent a high probability of elk use/kill, whereas lighter shaded areas represent a low probability of elk use/kill.

Distance to protected area (RMNP and DMPP&F) was the most important covariate (Table 1 and 2A [average AIC_*w*_ = 1.00]) influencing resource selection patterns of collared elk for both sexes. Juvenile males selected areas closer to protected areas (β_park_ = −27.26) compared with collared adult females (β_park_ = −15.28, Fig. 2A; standardized parameter estimates are presented in Supporting Information, Table S2). Of the a priori models (Table 1), Model 1 (AIC_*w*_ *=* 0.99) appeared to be the best model for adult females compared with Model 2 (AIC_*w*_ *=* 0.56) for juvenile males. The most notable difference between these models was local-level forest within the quarter section, which was only present in the juvenile male model. Model-averaged results showed the most important covariates influencing adult female selection were distance to protected area, highways and towns, landscape-level forest cover, and crop cover (cumulative Akaike weight > 0.50; Table 2A). For juvenile males, the most important covariates were distance to protected area and towns, local- and landscape-level forest cover, road density, and cover types of crop and water (cumulative AIC_*w*_ > 0.50). Females avoided landscape-level forest, high road density, towns and highways (in this case, a positive estimate indicates avoidance of towns and highways), water and crop cover, and selected local-level forest (Fig. 2A), whereas juvenile males showed strong aversion to landscape-level forest, high road density, towns and water cover, and selected crop and local-level forest cover. Sexual differences, as determined from these resource selection coefficients, were apparent (Fig. 3). The predictive accuracy using withheld model-testing data was (*r*_s_ = 0.72) for both collared adult females and juvenile males.

**Table 1:** Differences in Akaike information criterion (ΔAICc; with small-sample bias adjustment; Burnham and Anderson 2002) and AICc weights (*w*) for adult female and juvenile male candidate resource selection function models during the hunting season (September–February; 2002–2012) in southwestern Manitoba, Canada. AIC_c_*w* represents the probability of that model being the best in the candidate model set. Covariates are described in the Supporting Information, Table S1

	Collared females		Collared males		Killed females		Killed males	
				
Hypothesized models	ΔAICc	AIC_c_*w*	ΔAICc	AIC_c_*w*	ΔAICc	AIC_c_*w*	ΔAICc	AIC_c_*w*
**H**_**1**_ Park + Forestbuff + Highway + Town + Crop + Water	**0.00**	0.99	–	–	28.88	<0.001	18.18	<0.001
**H**_**2**_ Park + Forest + Forestbuff + Crop + Town + Water	30.10	<0.001	**0.00**	0.56	22.18	<0.001	20.13	<0.001
**H**_**3**_ Park + Road + Stream + Forestbuff + Town + Water	29.37	<0.001	4.81	0.05	3.95	0.10	**0.00**	0.69
**H**_**4**_ Park + Forest + Forestbuff + Road + Stream + Town + Water + Grassland	31.92	<0.001	0.91	0.36	**0.00**	0.72	2.59	0.19
**H**_**5**_ Park + Forest + Forestbuff + Road + Highway + Town + Crop + Water + Stream + Forage + Grassland + Wetland	8.94	0.01	6.41	0.02	5.16	0.05	7.27	0.02
**H**_**6**_ Park + Forest + Park ^*^ Forest + Road + Stream + Town + Water	32.05	<0.001	23.35	0.00	3.41	0.13	3.55	0.12
**H**_**7**_ Park + Forest + Forestbuff	61.73	<0.001	8.07	0.01	37.90	<0.001	29.62	<0.001
**H**_**8**_ Park + Forest + Park ^*^ Forest	61.80	<0.001	22.22	0.00	43.86	<0.001	39.25	<0.001
**H**_**9**_ Park	62.01	<0.001	24.88	0.00	52.98	<0.001	44.96	<0.001
**H**_**10**_ Forest	196.67	<0.001	75.39	0.00	89.29	<0.001	68.46	<0.001

**Table 2:** Cumulative AICc weights (*w*) for the covariates hypothesized to influence collared and hunter-killed adult female and juvenile male elk during the hunting season (September–February; 2002–2012) in southwestern Manitoba, Canada. All covariates with *w* > 0.5 are bolded. Cumulative AIC_c_ weight of a covariate is the percent of weight attributable to models containing that particular covariate and is calculated by summing the AIC_c_ weights of every model containing that covariate. Covariates are described in the Supporting Information, Table S1. Highway was removed from the full model for collared juvenile males due to multicollinearity

Covariate	Female elk	Male elk	Average Akaike cumulative weights, *w*_*i*_	Δ Akaike cumulative weights, *w*_*i*_
(A) Collared adult females and juvenile males				
Park	**1.00**	**1.00**	**1.00**	0.00
Forestbuff	**1.00**	**1.00**	**1.00**	0.00
Highway	**1.00**	–	–	–
Town	**1.00**	**1.00**	**1.00**	0.00
Crop	**1.00**	**0.68**	**0.64**	0.73
Forest	0.36	**1.00**	**0.68**	0.64
Road	0.48	**0.64**	**0.56**	0.16
Water	0.50	**0.67**	**0.59**	0.17
(B) Hunter-killed adult females and juvenile males				
Park	**1.00**	**0.96**	**0.98**	0.02
Road	**1.00**	**1.00**	**1.00**	0.00
Water	**1.00**	**1.00**	**1.00**	0.00
Stream	**1.00**	**1.00**	**1.00**	0.00
Town	**1.00**	**1.00**	**1.00**	0.00
Forestbuff	**0.75**	**0.73**	**0.74**	0.02
Grassland	**1.00**	0.18	**0.59**	0.82
Forest	**0.68**	0.35	**0.52**	0.33
Highway	0.32	**0.60**	0.46	0.28
Wetland	0.45	0.40	0.43	0.05

**Figure 4:**
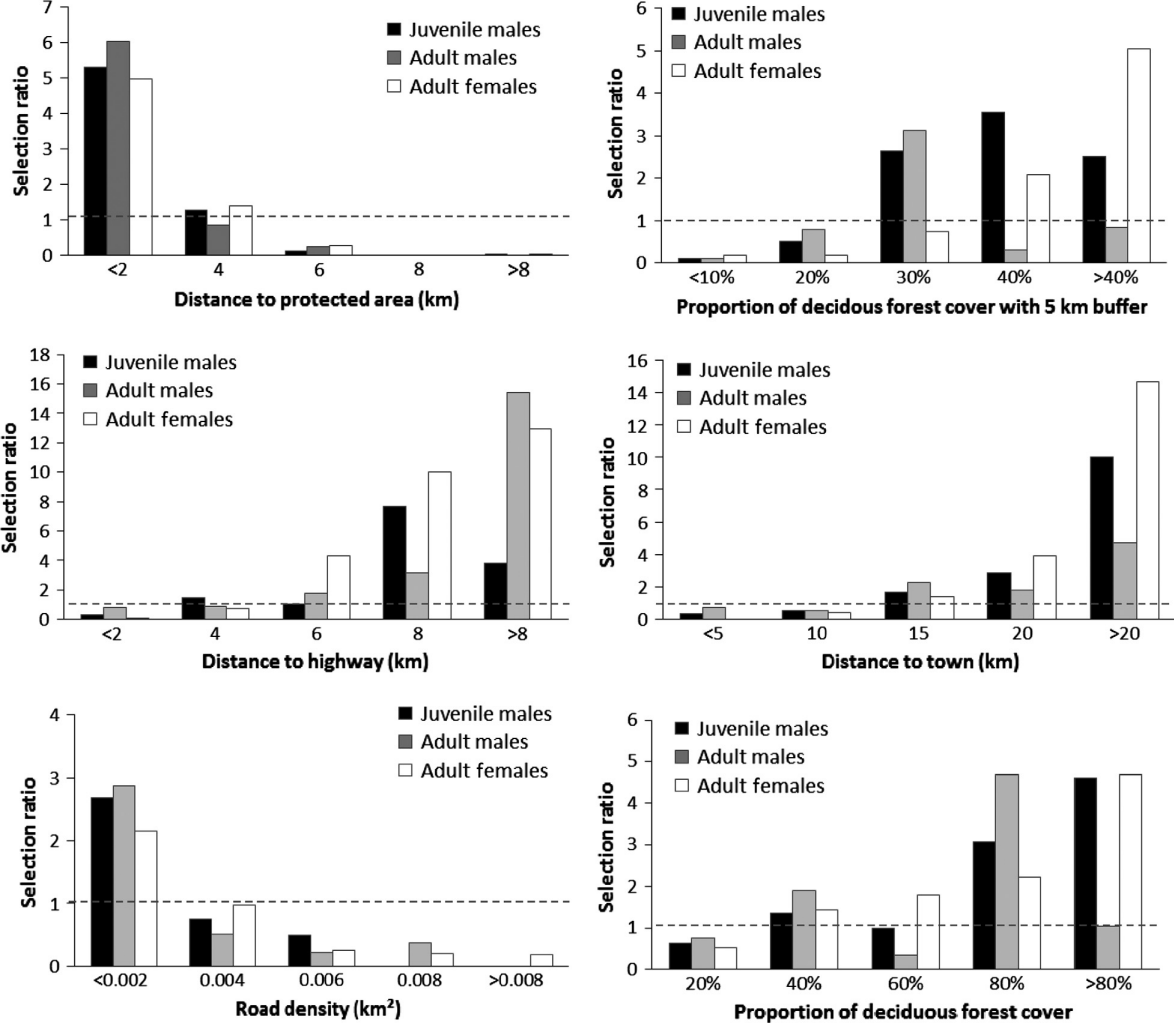
Selection ratios for the six covariates identified as most important to collared elk from the resource selection function models during the hunting season (September–February; 2002–2011) in southwestern Manitoba. Values > 1 indicate use is greater than availability, whereas values < 1 indicate use is less than availability.

Collared adult males had the highest SR for areas close to the parks (SR = 6.01, <2 km), compared with juvenile males (SR = 5.29) and adult females (SR = 4.96; Fig. 4). With a decrease in distance from the parks from 4 km to <2 km, the SR increased (five times for adult females and juvenile males, six times for adult males). Collared adult males also had the highest SR for areas with the lowest road density (SR = 2.87, <0.002 km^2^), compared with juvenile males (SR = 2.69) and adult females (SR = 2.13). A decrease in road density from 0.006 km^2^ to <0.002 km^2^ increased the SR for all cohorts by approximately 5 times. The SR increased most dramatically (average of 30 times) with increasing distance to highways from <2 km to >8 km for adult females and juvenile males, and 16 times for adult males.

#### Hunter-killed elk

Kill sites for both sexes were a function of multiple habitat covariates. The candidate a priori models (Table 1) identified Model 4 (AIC_*w*_ *=* 0.72) to be the best model for predicting adult female kill sites and Model 3 (AIC_*w*_ *=* 0.69) for juvenile male kills. Hunter-kill sites for both sexes were primarily driven by distance to protected area (Table 1 and 2B [average AIC_*w*_ = 1.00]), although the strength differed between the sexes (male β_park_ = −1.57, female β_park_ = −1.86, Fig. 2B; standardized parameter estimates are presented in Supporting Information, Table S2). The most notable difference between adult females and juvenile males was local-level forest and grassland cover, which was only present in the female model. Model-averaged results showed multiple covariates to be important for predicting kill sites for both sexes (AIC_*w*_ > 0.5, Table 2B). Adult females and juvenile males were killed closer to parks and streams and in sections with high local- and landscape-level forest cover. Both groups were killed away from sections with high road density, wetlands, grassland and water cover, highways and towns (positive estimate for towns and highways indicates avoidance; Fig. 2B). The predictive accuracy using withheld model-testing data was (*r*_s_ = 0.87) for adult females and (*r*_s_ = 0.82) for juvenile males.

**Figure 5:**
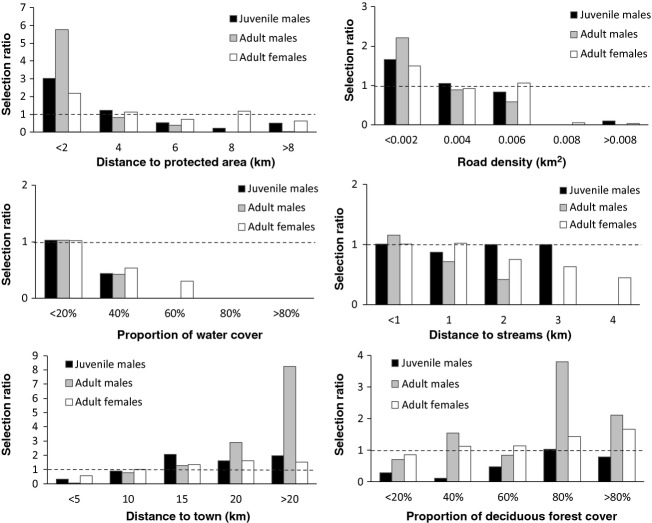
Selection ratios for the six covariates identified as most important for hunter-killed elk from the resource selection function models during the hunting season (September–February; 2003–2012) in southwestern Manitoba. Values > 1 indicate use is greater than availability, whereas values < 1 indicate use is less than availability.

The SR's for hunter-killed elk showed adult males to be killed in very different habitats compared with adult females and juvenile males (Fig. 5). For example, adult males were killed much closer to parks (SR = 5.75, <2 km) compared with adult females (SR = 2.19) and juvenile males (SR = 3.02), and a decrease in distance to the parks from 6 km to <2 km increased the SR most dramatically for adult males by approximately 12 times. Adult males were also killed in areas with low road density (SR = 2.21, <0.002 km^2^), furthest from towns (SR = 8.23, >20 km) and in heavily forested areas (SR = 3.82, 80% local forest cover). The SR changed most dramatically in adult males for both distance to town and proportion of local forest cover (an increase from 10 km to >20 km from towns increased the SR 8 times; an increase in local forest cover from <20% to 80% increased the SR 8 times).

#### Disease risk management areas

**Figure 6:**
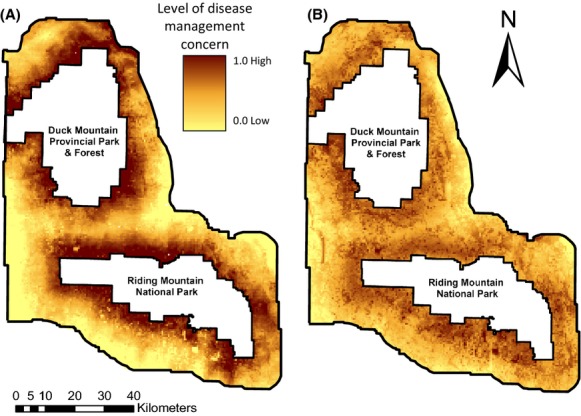
Predictive maps highlighting disease risk management areas for (A) juvenile male and (B) adult female elk, which largely reflect areas where they are known to strongly select but are not hunted effectively in southwestern Manitoba, Canada. Varying levels of disease management concern were obtained by calculating the difference in resource selection function scores between elk occurrence (Fig. 3A and B) and hunter-kill sites (Fig. 3C and D). Darker shaded sections represent areas where managers should redirect hunter distribution more effectively.

The distribution of areas for disease risk concern is more varied for juvenile males compared with adult females (Fig. 6). The most important areas were close to the park borders and within the remnant forest corridors between the parks. Disease risk areas for adult females are more uniform, with the most important areas at northwestern RMNP and western DMPP&F.

## Discussion

Few applications of human hunting to disease management attempt to target hunting at fine spatial scales or demography. However, there are important implications for disease management of sex- and age-specific differences in resource selection and kill sites, as we demonstrate. We present a novel approach using widely available data to quantify and map high-risk areas for disease transmission where hunting can be reallocated in an effort to improve disease monitoring and control. This strategy creates important opportunities to take a proactive approach to help limit disease spread, where management programs are based on contemporary analytical tools such as GIS that enhance our ability to evaluate the relationship between resource selection and mortality by predators.

Human and natural predators are known to create a landscape of fear, causing individuals to remain in or close to heavily forested refuge areas for protection (Swenson 1982; Burcham et al. 1999; Hernández and Laundré 2005). Hunters can have similar or even stronger effects on animal behavior compared with natural predators (Ciuti et al. 2012b). Indeed, Gude et al. (2006) and Proffitt et al. (2009) found that hunting constituted a greater effect on behavior of elk, such as grouping and distribution, than did naturally occurring predators such as wolves. Our results indicate that adult females made more use of the agriculture-dominated landscape during the hunting season and were killed further from the park boundaries compared with males. The majority of collared and killed males (particularly adult males) were found much closer to the park boundaries and therefore may perceive greater hunter risk compared with females. It is also possible that during the rut, which coincides with part of the hunting season, males near the park boundaries allocate more time to mating-related behavior rather than foraging or vigilance (Bowyer 1981; Wolff and Horn 2003). This type of muted antipredator response may render individuals more vulnerable to hunters (Neumann et al. 2009), particularly younger males (<4 years), which may be a result of inexperience compared with mature adult males (Wolf et al. 2009).

Distance to protected area was the most influential covariate of elk resource selection and hunter-kill sites for each cohort during the hunting season; however, other environmental factors also had a modulating effect. Both sexes selected local-level forest cover, presumably for protection from predators (Swenson 1982; Ciuti et al. 2012a), although selection was stronger for males, particularly adult males. The availability of agricultural crops was also an important factor for collared elk during the hunting season. Because open habitats are prime locations for hunters to detect and kill animals, we expected that there would be more kills; however, we did not find cropland to be an important predictor of a kill site. Indeed, agricultural crops were still strongly avoided by collared elk during the hunting season. Previous studies have found ungulates to move onto private agricultural land as an alternative form of security to minimize encounters with hunters (Burcham et al. 1999; Conner et al. 2001), as hunting is typically not allowed on these lands. In contrast, hunting is permitted on 70% of privately owned land with permission of the landowner in our study area (Brook 2008).

High road density has been shown to reduce local hunter success of elk over time, either due to increased hunting pressure causing animals to avoid these areas (Gratson and Whitman 2000) or increased access by hunters (Brinkman et al. 2007). Our results suggest that both sexes were killed in areas with relatively low road density in this region, suggesting hunters will expend more effort and are successful in habitat types with few or no roads (Lebel et al. 2012). Elk avoided areas with heavy road traffic during the hunting season, suggesting that elk associate roads with increased hunter risk and disturbance (Unsworth et al. 1993). Collared males (both age classes) showed stronger aversion to roads compared with collared adult females, which also concurs with findings from McCorquodale et al. (2003).

Despite demography playing a central role in the allocation of hunting licenses and that disease in wildlife is rarely uniform in the prevalence at which it occurs in different cohorts, combining the two to limit disease spread is uncommon. A practical application of this synthesis would be to target adult males (Schmitt et al. 2002; Grear et al. 2006), which have the highest prevalence of bovine TB in the area (Shury and Bergeson 2011). More specifically, focused hunts for males should occur in habitats that are regularly used but currently underrepresented in the distribution of kill sites. For example, adult males rarely leave RMNP and use very specific habitats, such as those with 80% forest cover and low road density (<0.002 km^2^) that are directly adjacent to the RMNP boundary. As such, targeting this highest risk cohort requires hunting to be concentrated in areas within <2 km from the RMNP border. For adult females and juvenile males, hunters should also focus in areas with low road density (<0.002 km^2^) but in areas further from the park boundary (within 10 km), particularly for adult females, because they move further from the park and are not hunted as effectively.

Increased hunting pressure along the borders of parks should continue to generate a landscape of fear for elk and remove bold animals venturing far out from protected areas. This pressure would ultimately restrict animals and disease to RMNP. Efforts should also be made to improve hunters' efficiency (Lebel et al. 2012) and ensure that the distribution of hunters better matches with animal distribution to avoid creating areas with little or no hunting pressure where animals can cluster and survive (e.g., Burcham et al. 1999; Conner et al. 2001).

The aim of sustainable wildlife management is to design a hunting policy that simultaneously optimizes population sex and age structure, elk density, and hunter-kill levels in the face of increasing pressures such as changing habitat conditions and disease threat. However, few management plans adequately account for the threat of disease in an area such as Manitoba where endemic bovine TB and emerging CWD threaten resident ungulates. It is crucial to understand the relationship between resource selection patterns and kill sites of hosts to predict disease risk and create opportunities to redirect hunting for disease control as shown here. Our disease risk management maps provide a complementary tool that allows managers to precisely evaluate hunter success, creating opportunities to redirect the distribution of hunters and improve disease testing. Indeed, a better understanding of the underlying mechanisms that drive the distribution and abundance of hosts and their hunters would also be an important prerequisite for appropriate disease monitoring and control.

## Supporting Information

**Table S1.** A priori selection of environmental covariates for elk (*Cervus canadensis manitobensis*) to derive resource selection function models and selection ratios at the scale of the quarter section (*n* = 20,970) during the hunting season (September–February) in southwestern Manitoba, Canada.

**Table S2**. Standardized parameter estimates from logistic regression resource selection function models for collared and hunter-killed adult females and juvenile males during the hunting season (September–March; 2002–2012) in southwestern Manitoba, Canada. Model-averaged *β* coefficient for each predictor covariate derived from an average of all possible models with a change in Akaike value < 2 (ΔAIC, 2.0). Covariates are described in Supporting Information, Table S1.

**Figure S1.** Overall schematic outline of the analyses to map disease risk management areas.
