# Supplementary material for: Targeting hunter distribution based on host resource selection and kill sites to manage disease risk
Source: Ecol Evol. 2013 Oct 1;3(12):4265–77. doi: 10.1002/ece3.788 (PMC3853570; doi:10.1002/ece3.788)

Supporting Information

Table S1. *A priori* selection of environmental covariates for elk (*Cervus canadensis manitobensis*) to derive resource selection function models and selection ratios at the scale of the quarter section (*n* = 20,970) during the hunting season (Sep-Feb) in southwestern Manitoba, Canada.

Covariate	Mean ± S.E.	Description of covariate
Park	1.67 ± 0.01	Minimum distance to protected area or refuge (RMNP and DMPP&F; km)
Forestbuff	0.20 ± 0.01	Proportion of deciduous forest cover within a 5 km buffer around quarter section
Town	9.08 ± 0.12	Minimum distance to town (km)
Highway	2.32 ± 0.03	Minimum distance to highway (km)
Forest	0.18 ± <0.001	Proportion of deciduous forest cover within the quarter section
Road	0.01 ± <0.001	Density of roads (km^2)
Crop	0.44 ± 0.01	Proportion of annual cereal/oilseed
Stream	0.79 ± 0.01	Minimum distance to stream (km)
Water	0.03 ± <0.001	Proportion of water cover including lakes and rivers
Forage	0.04 ± <0.001	Proportion of perennial forage crops
Wetland	0.02 ± <0.001	Proportion of marsh and fen
Grassland	0.25 ± <0.001	Proportion of grassland

Table S2. Standardized parameter estimates from logistic regression resource selection function models for collared and hunter-killed adult females and juvenile males during the hunting season (September–March; 2002–2012) in southwestern Manitoba, Canada. Model-averaged β coefficient for each predictor covariate derived from an average of all possible models with a change in Akaike value < 2 (ΔAIC, 2.0). Covariates are described in Supporting Information, Table S1.

	**Collared Females**	**Collared Males**	**Hunter-killed Females**	**Hunter-killed Males**
**Covariate**	β Coefficient	β Coefficient	β Coefficient	β Coefficient
Park (km)	-1.35	-2.70	-0.74	-0.68
Forest (%)	0.49	0.87	0.78	0.35
Forestbuff (%)	-0.61	-0.87	0.73	0.67
Highway (km)	1.39	--	0.33	0.50
Road (km^2)	-0.03	-0.22	-0.73	-0.74
Town (km)	0.91	0.77	0.99	1.44
Grassland (%)	--	--	-0.39	-0.13
Forage (%)	--	--	--	--
Crop (%)	-0.09	0.60	--	--
Water (%)	-0.04	-0.13	-2.13	-1.96
Wetland (%)	--	--	-0.55	-0.47
Stream (km)	--	--	1.44	1.49

Figure S1. Overall schematic outline of the analyses to map disease risk management areas.

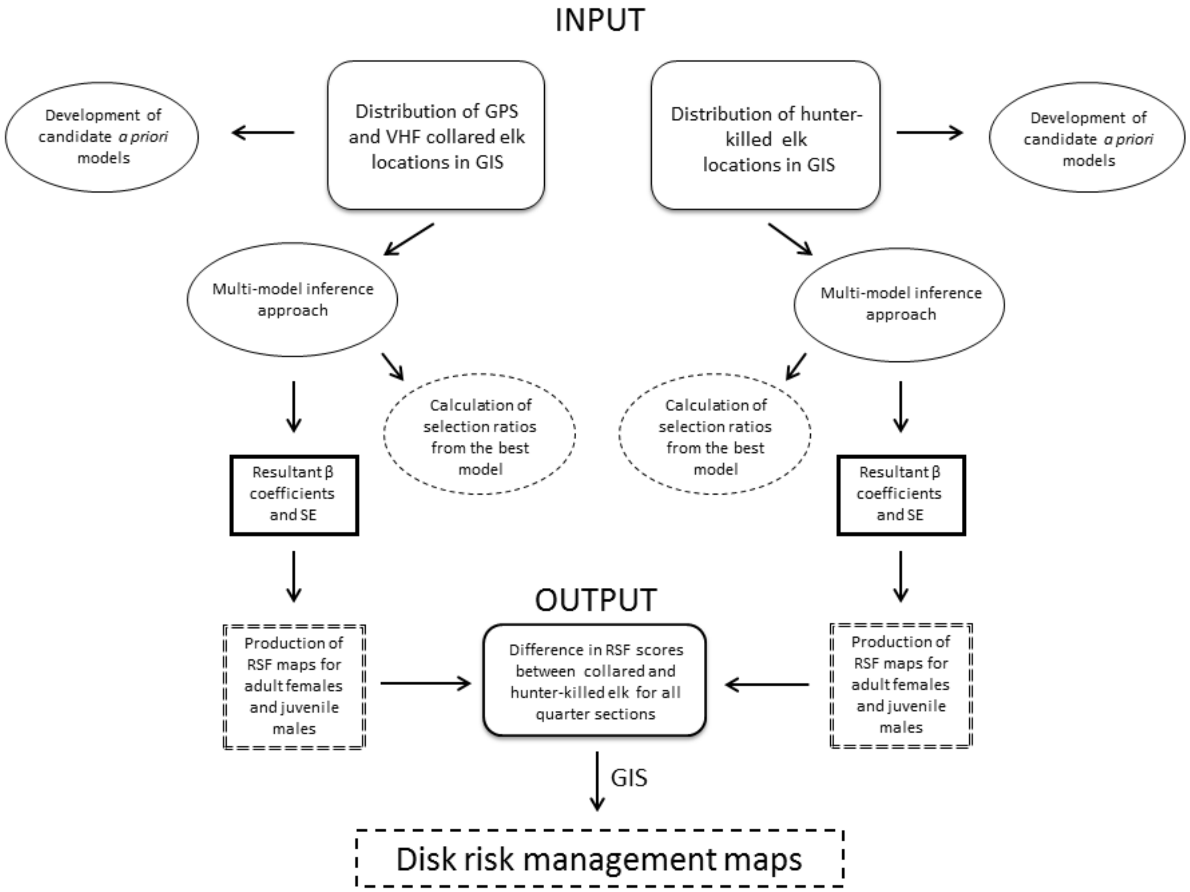

Supplement: Supplementary file 1 [file ece30003-4265-SD1.pdf]
